# Production of recombinant human annexin V by fed-batch cultivation

**DOI:** 10.1186/1472-6750-14-33

**Published:** 2014-04-27

**Authors:** Laura S Marder, Juleane Lunardi, Gaby Renard, Diana C Rostirolla, Guilherme O Petersen, José E S Nunes, Ana Paula D de Souza, Ana Christina de O Dias, Jocelei M Chies, Luiz A Basso, Diógenes S Santos, Cristiano V Bizarro

**Affiliations:** 1 Centro de Pesquisas em Biologia Molecular e Funcional (CPBMF), Instituto Nacional de Ciência e Tuberculose (INCT-TB), Pontifícia Universidade Católica do Rio Grande do Sul (PUCRS), Av. Ipiranga 6681, 90619-900 Porto Alegre, Brazil; 2 Programa de Pós-Graduação em Biologia Celular e Molecular, PUCRS, Porto Alegre 90619-900, Brazil; 3 Programa de Pós-Graduação em Medicina e Ciências da Saúde, PUCRS, Porto Alegre 90619-900, Brazil; 4 Quatro G Pesquisa & Desenvolvimento, LTDA, Porto Alegre 90619-900, Brazil; 5 Instituto de Pesquisas Biomédicas (IPB), Laboratório de Imunologia Molecular, PUCRS, Porto Alegre 90619-900, Brazil

**Keywords:** Recombinant human annexin v, Fed-batch cultivation, Large-scale, Apoptosis detection

## Abstract

**Background:**

Annexin V, a 35.8 kDa intracellular protein, is a Ca^+2^- dependent phospholipid binding protein with high affinity to phosphatidylserine (PS), which is a well-known hallmark of apoptosis. Annexin V is a sensitive probe for PS exposure upon the cell membrane, and used for detection of apoptotic cells both *in vivo* and *in vitro*. Large-scale production of recombinant human annexin V is worth optimization, because of its wide use in nuclear medicine, radiolabeled with ^99m^Tc, for the evaluation of cancer chemotherapy treatments, and its use in identification of apoptotic cells in histologic studies. Here we describe the high-yield production of a tag-free version of human annexin V recombinant protein by linear fed-batch cultivation in a bioreactor.

**Results:**

We cloned the human *ANXA5* coding sequence into the pET-30a (+) expression vector and expressed rhANXA5 in batch and fed-batch cultures. Using *E. coli* BL21 (DE3) in a semi-defined medium at 37°C, pH 7 in fed-batch cultures, we obtained a 45-fold increase in biomass production, respective to shaker cultivations. We developed a single-step protocol for rhANXA5 purification using a strong anion-exchange column (MonoQ HR16/10). Using these procedures, we obtained 28.5 mg of homogeneous, nontagged and biologically functional human annexin V recombinant protein from 3 g wet weight of bacterial cells from bioreactor cultures. The identity and molecular mass of rhANXA5 was confirmed by mass spectrometry. Moreover, the purified rhANXA5 protein was functionally evaluated in a FITC-annexin V binding experiment and the results demonstrated that rhANXA5 detected apoptotic cells similarly to a commercial kit.

**Conclusions:**

We describe a new fed-batch method to produce recombinant human annexin V in large scale, which may expand the commercial utilities for rhANXAV to applications such as *in vivo* imaging studies.

## Background

Annexin V, formerly known as human placental anticoagulation protein, is a member of a family of calcium-dependent phospholipid binding proteins [1] that binds preferentially to phosphatidylserine (PS), a negatively charged phospholipid highly enriched in the inner leaflet of plasma membranes [2]. Annexin V shows minimal capacity to bind phospholipids that are constitutively present in the outer leaflet of plasma membranes, such as phosphatidylcholine and sphingomyelin [1]. During the early stages of apoptosis, cells expose PS on the surface, which is specifically recognized by phagocytic cells [3]. The exposed PS residues can be bound selectively by annexin V with nanomolar to picomolar affinity [4,5]. Because of the role of apoptosis in the pathophysiology of many diseases, there is a wide range of current and potentially new applications for annexin V as an apoptotic marker in clinical diagnosis [6]. Staining cells simultaneously with annexin V labeled with the fluorochrome fluorescein isothiocyanate (FITC) and the non-vital dye propidium iodide (PI) allows the discrimination among intact cells (FITC^−^/PI^−^) early apoptotic cells (FITC^+^/PI^−^) and late apoptotic or necrotic cells (FITC^+^/PI^+^) [7]. Moreover, recombinant human annexin V (rhANXA5) serves as an important *in vivo* diagnostic tool when labeled with different radionuclides, such as iodine-123 (^123^I) and the metastable isotope technetium-99 (^99m^Tc) providing a broad range of imaging applications in apoptosis research, as single-photon emission computed tomography and auto-radiography to positron emission tomography [8]. In nuclear medicine, annexin V radiolabeled with ^99m^Tc or ^123^I is used to evaluate the efficacy of cancer therapy and disease progression or regression [9].

Because of its widespread use as a diagnostic tool, rhANXA5 is commercially produced in microorganisms such as *Escherichia coli* using recombinant DNA techniques [10-13]. To produce rhANXA5 in large scale in this study, we used *E. coli*, the most commonly used host for recombinant protein production [14]. In shake flask cultures, all components are added at the start of the cultivation, without the need to monitor or control any parameter such as pH or the level of dissolved oxygen, leading to slow growth and low recombinant protein production [15]. High cell-density culture techniques have been developed to improve productivity and to provide advantages such as reduced culture volume, enhanced downstream processing, reduced wastewater, lower production costs and reduced investment in equipment [14]. Fed-batch cultivation is an effective and simple method [16], allowing substantial concentrations of glucose, an inexpensive and readily usable carbon and energy source [17]. To our knowledge, there is currently no protocol available for a bioreactor-based, large-scale production of rhANXA5.

In this work, we describe a procedure for the production of 28.5 mg of homogeneous, nontagged rhANXA5 from 3 g wet weight of bacterial cells from bioreactor cultures. The identity and absence of host contaminants in purified rhANXA5 was confirmed by LC-MS/MS peptide mapping experiments. The molecular mass determination of intact rhANXA5 confirmed the integrity of the purified protein. Additionally, the produced rhANXA5 protein was shown to be functional in a bioassay for *in vitro* apoptosis/necrosis detection, in which it performed similarly to a commercially available kit.

## Results and discussion

### Cloning and expression of rhANXA5

The gene encoding human Annexin V, *ANXA5*, is located on human chromosome 4q27 locus and spans a region of DNA 29 kb in length containing 13 exons and 12 introns. *ANXA5* encodes a 35.8 kDa protein of 320 amino acid residues in length which is translated from a single mature transcript of approximately 1.6 kb [18]. The human *ANXA5* coding sequence was subcloned into the pET-30a (+) expression vector to generate the recombinant pET-30a (+)::ANXA5 plasmid. Both the sequence and the absence of PCR-introduced mutations in the *ANXA5* coding sequence were confirmed by automated sequencing.

The BL21 (DE3) and C41 (DE3) *E. coli* strains were transformed with the pET-30a (+)::ANXA5 construct by electroporation. The expression profiles were tested in lysogeny broth (LB) and in our semi-defined (SD) medium. The best results of soluble recombinant protein production from shaker cultivation were obtained using the BL21 (DE3) strain in SD medium induced with 1 mM IPTG at 37°C (Additional file 1: Figures S1 and Figure S2).

### Bioreactor cultivation

The best growth conditions found in shaker cultivations were applied to the bioreactor batch and fed-batch cultures. We employed our SD medium and a temperature of 37°C in all experiments.

Transformed BL21 (DE3) *E. coli* was grown from a master cell bank (MCB) under batch cultivation in 1 L of SD medium at 37°C monitoring glucose consumption. Within 4 h, glucose was depleted from media, indicating this was the right time to start feeding. We compared DO-stat, pH-stat and linear ascending feeding strategies in uninduced cultures. A biomass concentration of 26.01 g (DCW) L^−1^ was attained in an uninduced culture using the linear ascending feeding profile [17]. Lower values for biomass concentration were obtained using DO-stat (20.15 g (DCW) L^−1^) or pH-stat (11.59 g (DCW) L^−1^) feeding strategies. Therefore, we selected the linear ascending feeding strategy for further fed-batch cultivations.

**Figure 1:**
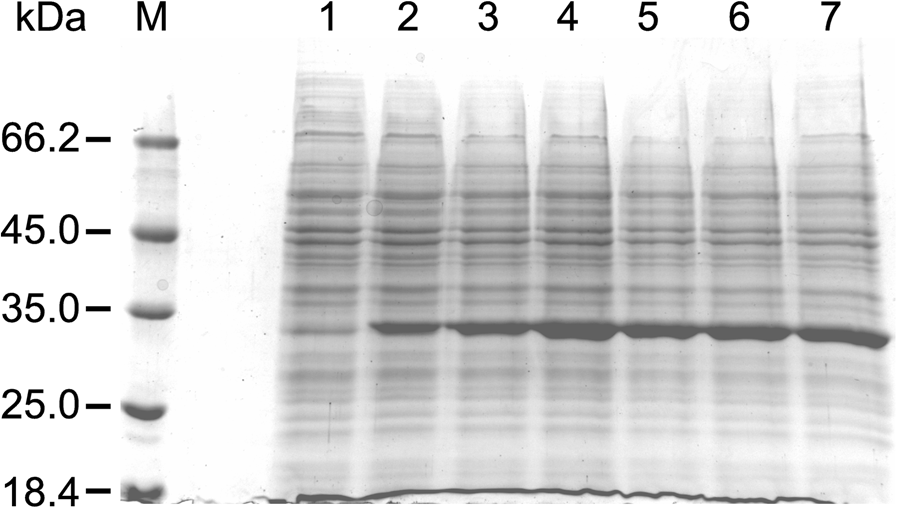
**SDS-PAGE analysis of rhANXA5 expression in fed-batch cultivations.** Recombinant *E. coli* BL21(DE3) cells were cultivated in semi-defined (SD) media for 30 h in fed-batch cultivations. Feeding started 4 h after the beginning of batch cultivation and rhANXA5 expression was induced after 18 h of cultivation by the addition of 1 mM IPTG to the cultures. M: Thermo Scientific™ Unstained Protein MW Marker; lane 1: sample collected immediately before IPTG induction (18 h culture); lanes 2–7: samples collected after IPTG induction from 20 h (lane 2), 22 h (lane 3), 24 h (lane 4), 26 h (lane 5), 28 h (lane 6), and 30 h (lane 7) cultures.

After 4 h of batch cultivation without feeding, the biomass concentration reached 4.71 g (DCW) L^−1^ (n = 4, SD = 0.6). We induced rhANXA5 expression in fed-batch cultivations with linear ascending feeding by adding IPTG to a final concentration of 1 mM. From three independent fermentations, we obtained a mean value of 27.48 g (DCW) L^−1^ (SD = 1.96) for the biomass concentration and a total protein content of 4.8 g L^−1^ (Figure 1). In contrast, we obtained a biomass concentration of 0.615 g (DCW) L^−1^ in shaker cultivation (OD_600nm_ = 1.84) after 6 h of culture. This represents a 45-fold increase in biomass concentration.

In an independent bioreactor cultivation, we performed densitometric analysis of culture samples (in triplicate) to calculate the product yield (g (rhANXA5) L^−1^), the productivity (g (rhANXA5) L^−1^ h^−1^) and the specific yield (g (rhANXA) g (DCW)^−1^). rhANXA5 corresponded to 40.6% (mean value) of the total protein content (n = 3, SD = 0.03) (Additional file 1: Figure S4). Therefore, we obtained a product yield of 1.95 g (rhANXA5) L^−1^ of culture medium, a specific yield of 0.0715 g (rhANXA5) g (DCW)^−1^ and a productivity of 0.065 g (rhANXA5) L^−1^ h^−1^, considering the entire fermentation period (30 h).

Moreover, we can consider the entire procedure, from bioreactor cultivation to protein purification, and calculate the yield and productivity of homogeneous rhANXA5. From four independent purifications, we obtained a mean value of 28.5 mg of purified rhANXA5 from 3 g wet weight of cells (n = 4, SD = 6.7). In terms of volumetric yield, we obtained (mean value) 0.983 g (purified rhANXA5) L^−1^ (n = 4, SD = 0.23). The productivity over the entire fermentation (30 h) was 0.0361 g (purified rhANXA5) L^−1^ h^−1^ (n = 4, SD = 0.0084).

### Purification

**Figure 2:**
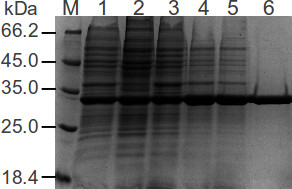
**SDS-PAGE analysis of rhANXA5 purification steps.** SDS-PAGE (12%) analysis of fractions from different steps of rhANXA5 purification. Each sample lane contains 8 μg of total protein. M corresponds to Thermo Scientific™ Unstained Protein MW Marker; lane 1: cells suspended in buffer A (50 mM Tris–HCl, pH 7.2, 10 mM CaCl_2_) with no previous centrifugation step; lane 2: supernatant of cells in buffer A after sonication and centrifugation (for 20 min, 24,400 × *g*, 4°C); lane 3: cell pellet from the previous centrifugation suspended in buffer B (50 mM Tris–HCl, pH 7.2, 20 mM EDTA); lane 4: suspension from previous step dialyzed against 2 L of 20 mM Tris–HCl, pH 8.0 for 3 times, overnight in the first step, followed by two steps of dialysis 2 h each; lane 5: supernatant after centrifugation of dialyzed sample (for 30 min, 24,400 × *g*, 4°C); lane 6: homogeneous preparation of annexin V eluted from the Mono Q column.

The overexpressed protein was purified by a single-step protocol consisting of a strong anion-exchange column (MonoQ HR16/10). Figure 2 shows the steps of rhANXA5 purification. The target protein eluted at approximately 190 mM of NaCl from a MonoQ HR 16/10 column (Additional file 1: Figure S3). The eluted protein was pooled and dialyzed against HEPES 100 mM NaCl pH 7.2, concentrated using an AMICON ultra-filtration membrane and stored at −80°C in 1 mL aliquots. This purification protocol yielded 28.5 mg of purified rhANXA5 from 3 g wet weight of cells (n = 4, SD = 6.7).

### rhANXA5 identification by mass spectrometry

Homogeneous rhANXA5 samples were desalted and digested with trypsin, and the peptide mixtures were analyzed in LC-MS/MS peptide mapping experiments. A total of 320 spectra were identified with 29 different peptides derived from rhANXA5 protein. These peptides covered 80% of the rhANXA5 sequence.

### Determination of rhANXA5 molecular mass

**Figure 3:**
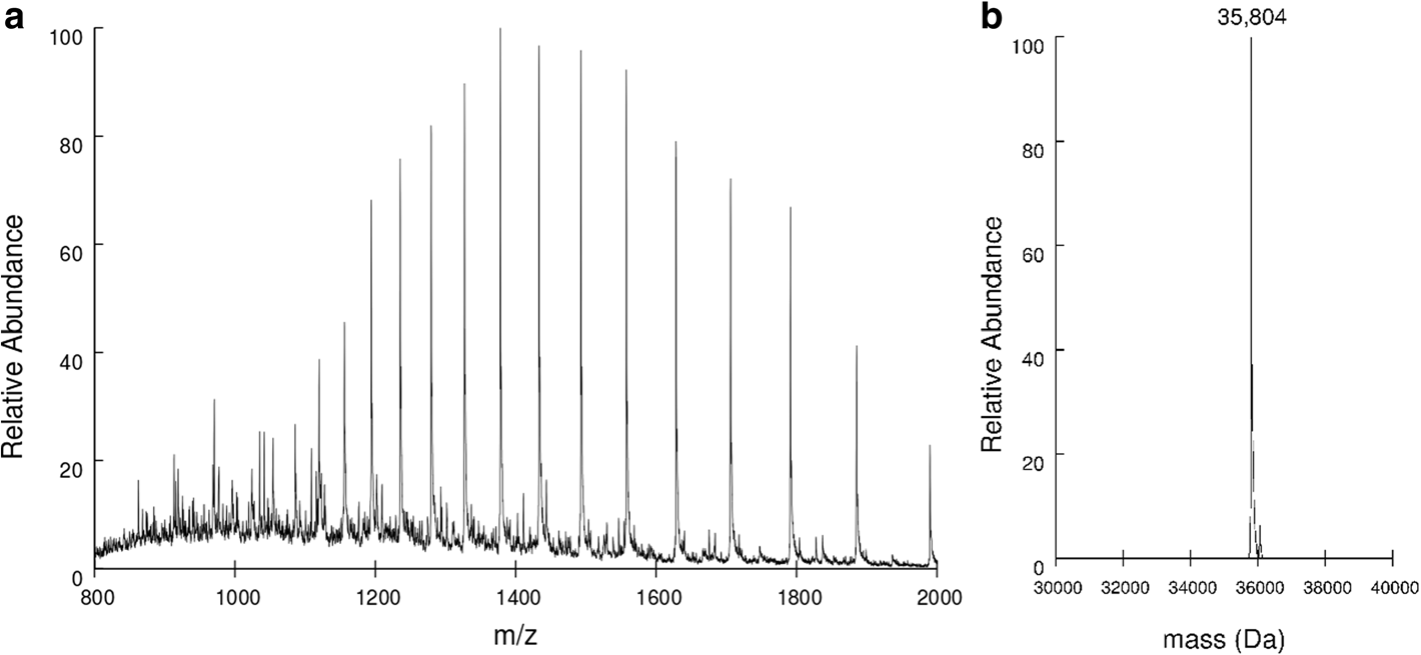
**Determination of rhANXA5 molecular mass by mass spectrometry. a)** ESI-FTMS spectra of rhANXA5 showing charge state distribution spanning from 18+ to 39+. **b)** Experimentally determined value of 35,804 Da for rhANXA5 average molecular mass after spectra deconvolution.

The spectra of intact rhANXA5 samples were recorded with a linear ion trap analyzer. Peaks spanning charge states 18+ to 39+ were detected (Figure 3a) and the spectra deconvoluted. We obtained a value of 35,804 Da for the average molecular mass of rhANXA5 (Figure 3b), consistent with the post-translational removal of the N-terminal methionine (theoretical average molecular mass of 35,937 Da with methionine and 35,805 Da without methionine).

### FITC-annexin V binding test

**Figure 4:**
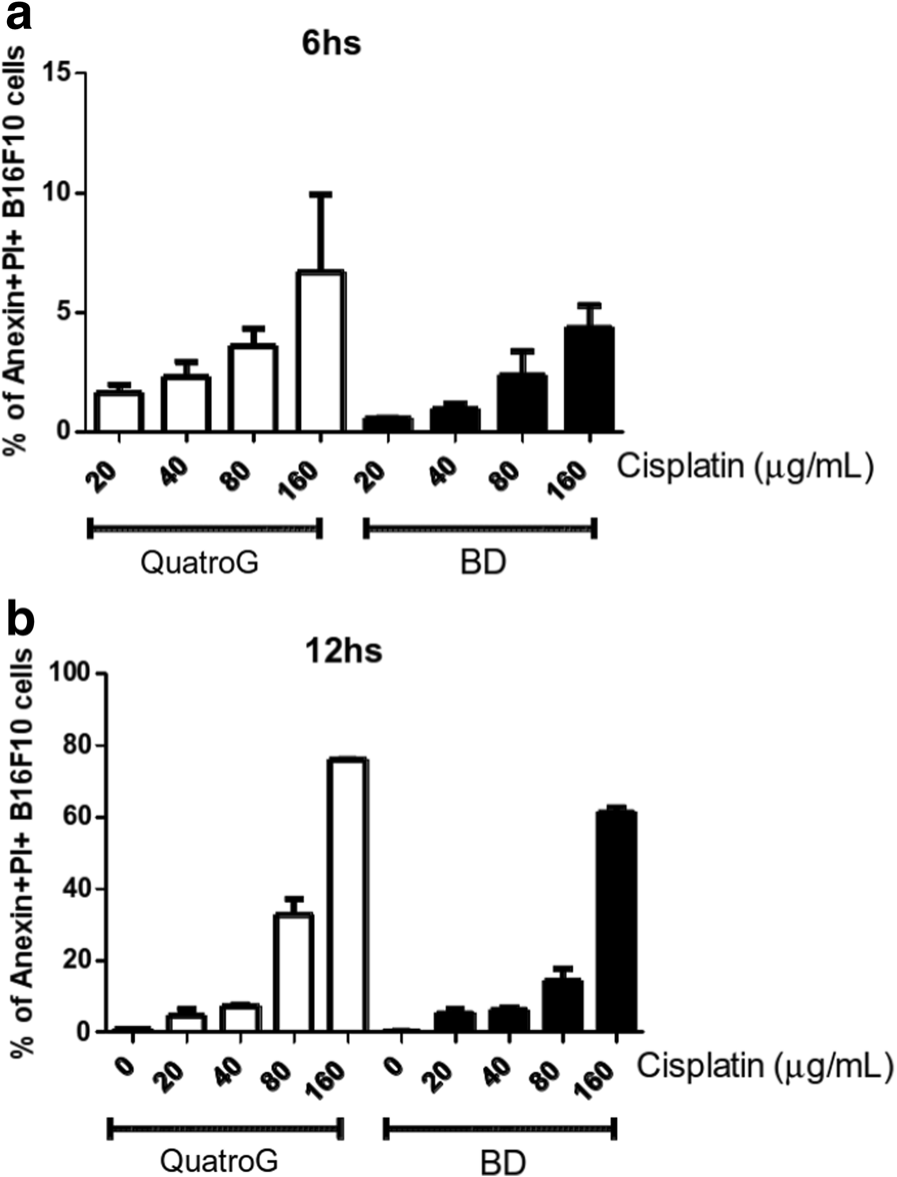
**Analysis of apoptosis in B16F10 cells.** Flow cytometric analysis of apoptotic cells stained with both FITC-labeled annexin V and PI using the BD Biosciences kit or a homemade kit (QuatroG) containing purified rhANXA5 conjugated with FITC. Apoptosis was induced by treating B16F10 cells with 20, 40, 80 or 160 μg/mL of cisplatin and double staining was performed after 6 h **(a)** or 12 h **(b)**.

**Figure 5:**
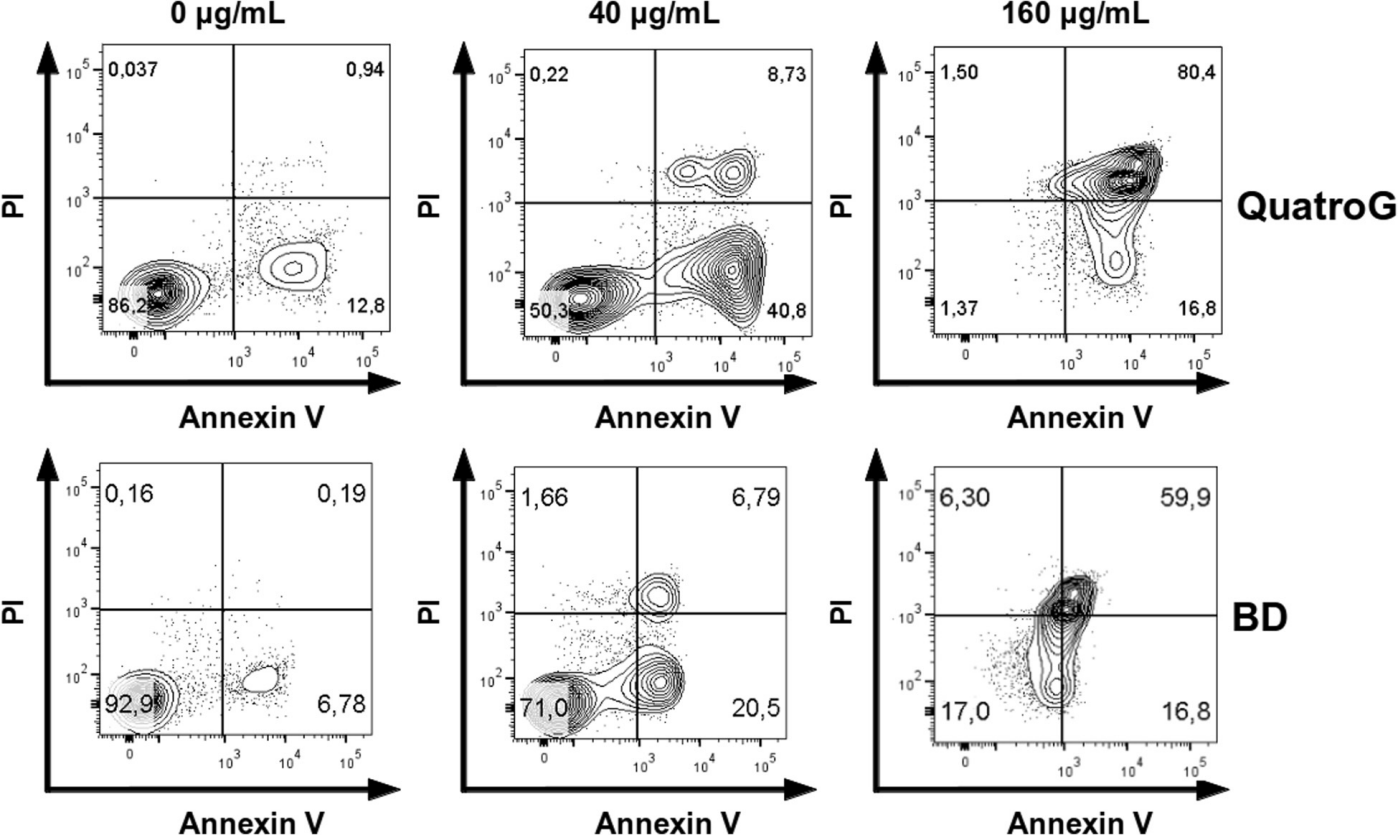
**Representative dot plots of flow cytometric analysis.** Dot plots of B16F10 cells stained with both FITC-labeled annexin V and PI using the BD Biosciences kit or a homemade kit (QuatroG) 12 h after inducing apoptosis with 0 μg/mL, 40 μg/mL or 160 μg/mL of cisplatin. The upper and lower right quadrants of each dot plot represents cells at late (PI and annexin V positive) and early (annexin V positive only) apoptosis.

To test the functional activity of purified rhANXA5, we assayed its ability to detect cells undergoing apoptosis. We treated B16F10 cells with cisplatin to induce apoptosis and after 6 or 12 h, we stained treated cells simultaneously with the non-vital dye PI and with rhANXA5 labeled with the fluorochrome FITC. Double staining allows the discrimination between intact cells (FITC^−^/PI^−^), early apoptotic cells (FITC^+^/PI^−^) and late apoptotic or necrotic cells (FITC^+^/PI^+^) [7]. The ability of FITC-labeled rhANXA5 to detect apoptotic cells was compared with a commercial kit from BD. We performed dose curve experiments with different concentrations of cisplatin and stained treated cells for 6 h (Figure 4a) or 12 h (Figure 4b) after treatment. We obtained similar results using our homemade kit (QuatroG) or the commercial kit at different times after treatment (6 h or 12 h) and concentrations of cisplatin (20, 40, 80 and 160 μg/mL), indicating that purified rhANXA5 is functionally active. Dot plot representations of flow cytometry experiments with cells stained 12 h after treatment clearly show differences in the distribution of FITC/PI cell populations between untreated cells (negative control), cells treated with 40 μg/mL and cells treated with 160 μg/mL of cisplatin (Figure 5).

## Conclusions

Fed-batch cultivations represent an alternative to shaker cultivations, allowing control of process variables and improvement on biomass concentration and product yields [15]. A chimeric protein containing the C-terminus of hirudin fused to annexin V was previously expressed in large scale using fed-batch fermentation [19]. However, to the best of our knowledge, this is the first report of a scale up of rhANXA5 production.

Recombinant protein production methods must continuously be improved to meet commercial demands [14,20]. In this work, we produced 28.5 mg of purified recombinant human annexin V from 3 g wet weight of cells (n = 4, SD = 6.7) obtained in fed-batch cultures induced with IPTG. Moreover, this protocol generated a yield of homogeneous rhANXA5 (mean value) of 0.983 g (purified rhANXA5) L^−1^ (n = 4, SD = 0.23). The productivity over the entire fermentation (30 h) was 0.0361 g (purified rhANXA5) L^−1^ h^−1^ (n = 4, SD = 0.0084).

rhANXA5 could be commercially distributed to several research groups and to radiopharmaceutical companies. The use of fluorescence-labeled annexin V to identify apoptotic cells is currently limited to histologic and cell-sorting studies performed *in vitro*. Its use for *in vivo* imaging studies is hampered mainly by cost issues. Hence, production in large scale may expand the commercial utilities for rhANXA5, reducing costs and allowing a greater access of the scientific and physician community to this product.

## Methods

### Strains and plasmids

The *E. coli* strains BL21(DE3) and C41(DE3) were purchased from Novagen® (EMD Biosciences, Inc., Madison, WI, USA) and Lucigen Corporation (Middleton, WI, USA), respectively. The PCR-Blunt® cloning vector was purchased from Invitrogen® (Carlsbad, CA, USA) and the pET-30a (+) expression vector was from Novagen®.

### Molecular cloning of rhANXA5

Oligonucleotides were designed based on the human *ANXA5* coding sequence in the GenBank (accession number: NM_001154.3) National Institute of Health (NIH) genetic sequence database [18]. Specific primers were designed to contain *Nde*I (forward primer: 5’ GCG *CAT ATG* GCA CAG GTT CTC AGA GGC ACT 3’) and *Hind*III (reverse primer: 5’ GCG *AAG CTT* TTA GTC ATC TTC TCC ACA GAG C 3’) restriction sites (italics). The human *ANXA5* gene sequence was PCR-amplified from a human blood cDNA sample.

The amplified *ANXA5* coding sequence was cloned into the PCR-Blunt® vector, cleaved with *Nde*I and *Hind*III restriction endonucleases (New England BioLabs®, Ipswich, MA, USA) and subcloned into the pET-30a (+) expression vector, previously digested with the same restriction enzymes, to generate the pET-30a (+)::ANXA5 plasmid. The cloned *ANXA5* sequence was confirmed by automated DNA sequencing.

### Media preparation

Lysogeny broth (LB) (tryptone, 10 g L^−1^; yeast extract, 5 g L^−1^; NaCl, 10 g L^−1^) was sterilized by autoclaving (30 min at 121°C). LB medium was used for shake-flask cultivation and for inoculum development of bioreactor cultivations.

Semi-defined medium (SD) [21] was used for shake-flask cultivations as well as fed-batch bioreactor cultivations, as both initial batch and also feeding media (with varied glucose and MgSO_4_ concentrations). SD medium contains 0.5 g L^−1^ NaCl (a), 1 g L^−1^ NH_4_Cl (b), 20 g L^−1^ yeast extract (c), 6 g L^−1^ Na_2_HPO_4_ (d), 3 g L^−1^ KH_2_PO_4_ (e), 1 μg L^−1^ thiamine (f), 1 mM MgSO_4_ (g), 0.1% trace solution (h), and 0.1 mM CaCl_2_ (i). For both shake-flask and bioreactor fed-batch cultivations (initial batch culture), SD medium was supplemented with glucose to a final concentration of 5 g L^−1^ (j). Components (a) to (e) were assembled and sterilized together by autoclaving (30 min at 121°C), while components (g) to (i) were sterilized separately by autoclaving and added under aseptic conditions. Thiamine solution (f) was filter-sterilized. Trace solution (h) contained 2.8 g L^−1^ FeSO_4_, 2 g L^−1^ MnCl_2_, 2 g L^−1^ CaCl_2_, 0.26 g L^−1^ CuCl_2_, and 0.3 g L^−1^ ZnSO_4_. For feeding medium in bioreactor cultivations, SD medium was supplemented with glucose (final concentration of 300 g L^−1^) and MgSO_4_ (40 mM). To prepare this feeding medium, we mixed equal parts of a 2x concentrated SD medium and a 600 g/L glucose stock solution, both sterilized separately. SD medium used in bioreactor cultivations was also supplemented with 100 μL of Antifoam 204 (Sigma-Aldrich, São Paulo, SP, Brazil) per liter of culture. Both LB and SD media were supplemented aseptically with filter-sterilized kanamycin to a final concentration of 30 μg mL^−1^.

### Shake-flask cultivation

*E. coli* BL21(DE3) and C41(DE3) strains were transformed with the pET-30a (+) vector or the pET-30a (+)::ANXA5 recombinant plasmid by electroporation. Transformant colonies were selected on LB agar plates containing 30 μg mL^−1^ kanamycin [22]. Isolated colonies were selected and grown overnight (at 37°C and 180 rpm) in 5 mL of LB supplemented with 30 μg mL^−1^ kanamycin. To compare the expression profile with different strains (BL21(DE) or C41(DE3)) or media (LB or SD medium), saturated cultures were inoculated in 50 mL of LB or SD medium for shake-flask cultivations and grown at 37°C and 180 rpm to an optical density (OD_600 nm_) of 0.4–0.6. At this growth stage, we induced rhANXA5 expression by adding isopropyl-β-D-thiogalactopyranoside (IPTG) to a final concentration of 1 mM. At 3, 6, 9 or 24 h after induction, cells were harvested by centrifugation (11,800 *g*) for 30 min at 4°C, and the pellet was stored at −20°C. The expression of the recombinant soluble protein was confirmed by 12% sodium dodecyl sulfate-polyacrylamide gel electrophoresis (SDS-PAGE) visualized by Coomassie® Brilliant Blue R-250 staining. For biomass measurements from shake-flask cultures, isolated colonies were selected and grown overnight (at 37°C and 180 rpm) in 10 mL of LB supplemented with 30 μg mL^−1^ kanamycin and inoculated in 500 mL of SD medium. Inoculated cultures were grown (37°C and 180 rpm) to an optical density (OD_600 nm_) of 0.4–0.6, rhANXA5 expression was induced by the addition of IPTG to a final concentration of 1 mM, and cells were harvested after 6 h of IPTG induction.

### Inoculum development for bioreactor cultivation

A master cell bank (MCB) of transformed *E. coli* BL21(DE3) cells containing the pET-30a (+)::ANXA5 recombinant plasmid was prepared in 50% glycerol and stored at −80°C. For inoculum development, 150 μL of *E. coli* BL21(DE3) MCB cells (stored at −80°C) were grown overnight at 180 rpm and 37°C in 1 L flasks containing 250 mL of LB medium supplemented with 30 μg mL^−1^ kanamycin. The final optical density (OD_600_) for each pre-inoculum culture was determined spectrophotometrically. For each experiment, we calculated and collected the initial volume of pre-inoculum culture needed to start bioreactor cultivation with an initial OD_600_ of 0.1. Collected aliquots were diluted in LB medium to a final volume of 100 mL before inoculation in 900 mL of SD medium for bioreactor cultivation.

### Bioreactor cultivation

Batch and fed-batch culture experiments were conducted in a BIOSTAT® B Plus bioreactor (Sartorius Stedim, Goettingen, Germany) with two 2 L stirred tanks, filled with 1 L of SD medium each, at 37°C, pH 7.0 and supplemented with kanamycin. For pH control, 12% (v/v) ammonium hydroxide and 10% (v/v) phosphoric acid were employed. The bioreactor was equipped with two Rushton turbines and with agitation, aeration, temperature and pH controllers. A polarographic electrode was used to measure the dissolved oxygen concentration (DOC) in the culture. The pO_2_, pH, stirrer speed, base and acid consumption and aeration rate were measured online and recorded by an external data acquisition and control system (Sartorius Stedim). Feeding was implemented using the bioreactor proprietary software micro-DCU system v. 0.63 (Sartorius Stedim) that allows controlling a peristaltic pump for feeding medium addition. The flow rate varied linearly from 0.066 mL min^−1^ (starting after 4 h of batch culture) to 0.594 mL min^−1^ after 26 h of feeding (30 h of bioreactor cultivation). In batch cultures, the DOC was maintained at 30% by cascading agitation (400–1000 rpm) with constant aeration rate (1vvm), and the process was finished when the biomass reached stationary phase.

Fed-batch cultivations were started as batch cultures with feeding starting at 4 h of cultivation (approximately OD_600nm_ 16.0) with SD as feeding medium (see subsection "Media preparation"). Different feeding strategies were tested. In DO-stat feeding fermentations, with DOC setpoint at 30%, the agitation rate was maintained at 800 rpm (after feeding initiation). In fed-batch fermentations with a linear ascending or pH-stat feeding, the DOC was maintained at 30% by cascading agitation (400–1000 rpm) with constant aeration rate (1 vvm).

For linear ascending feeding profile we used the following equation:

F=at+b

where *F* is the feeding rate (mL min^−1^), *t* the cultivation time after initiation of the fed-batch culture (min) and, *a* and *b* are constants for the linear ascending feeding profile [23].

### Analytical methods

Samples were withdrawn periodically for quantitative analysis along the cultivation. Cell growth was monitored by measuring the optical density at 600 nm (OD_600 nm_) in a spectrophotometer. One optical density unit was found to be equivalent to 0.3342 g L^−1^ of dry cell weight by gravimetric quantitation. Glucose concentration in the medium was measured with a glucose analyzer (model 2700 select, Yellow Springs Instruments, Yellow Springs, OH, USA). Acetate concentration was determined by high performance liquid chromatography (Äkta Purifier, GE HealthCare©, São Paulo, Brazil) equipped with an Aminex HPX-87H column (Bio-Rad Laboratories, Hercules, CA, USA), using 0.005 M H_2_SO_4_ as mobile phase and a UV-detector. The protein expression was analyzed by 12% SDS-PAGE stained with Coomassie® Brilliant Blue R-250 staining. The annexin V protein produced was quantified using the Qubit® Protein Assay Kit (Invitrogen™, Life Technologies, São Paulo, SP, Brazil) and a Qubit® 2.0 Fluorometer (Invitrogen™).

### Purification

ANXA5 recombinant protein was purified using a Fast Performance Liquid Chromatography (FPLC) ÄKTA Purifier System (GE HealthCare^©^). All chromatographic steps were carried out at 4°C. Sample elution was monitored by UV detection at 215, 254 and 280 nm and fractions were analyzed by 12% SDS-PAGE. According to a previous report [24], frozen cells (3 g wet weight) were suspended in 30 mL of buffer A (50 mM Tris HCl, 10 mM CaCl_2_ pH 7.2) and incubated with 1 mM of phenylmethanesulfonylfluoride for 30 min at 4°C. The cells were disrupted by sonication (eight pulses of 10”) and centrifuged at 38,900 *g* for 30 min. The supernatant was discarded and the pellet was completely dissolved in 30 mL of buffer B (50 mM Tris HCl, 20 mM EDTA pH 7.2), stirred for 30 min at 4°C, and clarified by centrifugation at 38,900 *g* for 30 min at 4°C. The supernatant was dialyzed against 20 mM Tris HCl pH 8.0 (3 × 2 L, 3 h each). Residual precipitate was removed by centrifugation (38,900 *g* for 20 min) and the supernatant was loaded on a MonoQ HR 16/10 anion exchange column (GE Healthcare) previously equilibrated with 20 mM Tris HCl pH 8.0. Protein was eluted with 25% linear gradient of 20 mM Tris HCl, 1 M NaCl pH 8.0 at 1 mL min^−1^ flow rate. Homogeneous rhANXA5 was eluted at approximately 190 mM NaCl. Fractions containing homogeneous rhANXA5 were pooled, dialyzed against 20 mM N-2-hydroxyethylpiperazyne-N’-2-ethanesulfonic Acid (HEPES), 100 mM NaCl pH 7.2 and concentrated using an AMICON (Millipore Corporation, Bedford, MA, USA) ultra-filtration membrane (MWCO = 10 kDa), and stored at −80°C. Protein concentration was determined with Qubit® Protein Assay Kit using a Qubit® 2.0 Fluorometer.

### rhANXA5 identification by mass spectrometry

rhANXA5 preparations (1 nmol) were desalted and subjected to proteolytic degradation using trypsin. The resulting peptides were separated by chromatography using 15 cm capillary columns (150 μm i.d., Kinetex C18 core-shell particles, Phenomenex, Inc., Torrance, CA, USA) and a nanoLC Ultra 1D plus equipment (Eksigent, Redwood City, CA, USA). Separated peptides were analyzed using an LTQ-Orbitrap hybrid mass spectrometer (Thermo Fisher Scientific Inc, Waltham, MA, USA). The chromatographic method used a step gradient from mobile phase A (0.1% formic acid in water) to mobile phase B (0.1% formic acid in acetonitrile): 0–2% B over 5 min; 2–10% B over 3 min; 10–60% B over 60 min; 60–80% B over 2 min; 80% B isocratic for 10 min; 80–2% B over 2 min; and 2% B isocratic for 8 min. We performed MS/MS fragmentation using collision-induced dissociation (CID) with an activation Q of 0.250, an activation time of 30.0 ms, and an isolation width of 1.0 Da. Using the Proteome Discoverer software (v. 1.3), we compared experimentally obtained MS and MS2 spectra with the *in silico* trypsin digestion of the human proteome. We allowed a precursor tolerance of 10 ppm, a fragment tolerance of 0.8 Da, static carbamidomethylation on cysteines, and oxidation on methionine residues. We restricted our analysis to matches with an Xcorr score > 2.0 for doubly charged ions and Xcorr score > 2.5 for triply charged ions.

### Determination of rhANXA5 molecular mass

Purified rhANXA5 samples were desalted, reconstituted in acetonitrile 50%/MilliQ-water 49%/formic acid 1% and directly injected using a 500 μL syringe (Hamilton Company, Reno, NV, USA) in a static mode into an IonMax electrospray ion source. The electrospray source parameters were as follows: positive ion mode, 4.5 kV of applied voltage to the electrospray source, 5 arbitrary units (range 0–100) of sheath gas flow, 45.6 V of capillary voltage, 250°C of capillary temperature, and 238.8 V of tube lens voltage. Full spectra (600–2000 m/z range) were collected on a Thermo Orbitrap Discovery XL in profile mode using the linear ion trap analyzer (ITMS mode). The average spectrum was processed with the software MagTran [25] for charge state deconvolution.

### FITC-annexin V binding test

To confirm the ability of rhANXA5 to detect cells undergoing apoptosis, we labeled rhANXA5 with the FluoroTag™ FITC Conjugation Kit (Sigma-Aldrich®). Our home-made kit also contained PI solution (Sigma-Aldrich®) and a 10 × binding buffer (0.1 M HEPES-NaOH pH 7.4, 1.4 M NaCl, 25 mM CaCl_2_). B16F10 melanoma cells (4X10^4^), a kind gift from Dr. Peter Henson (National Jewish Center for Immunology, Denver, CO, USA) were cultured in Dulbecco's Modified Eagle's Medium (DMEM) supplemented with 10% fetal bovine serum (FBS), gentamicin 80 mg L^−1^ (Novafarma, Anápolis, Brazil), and Fungisone 5 mg L^−1^ (Bristol Myers Squibb, New York, NY, USA). To induce apoptosis, the cells were treated with cisplatin (Libbs, Embu, SP, Brazil) at different concentrations (0, 40, 80 and 160 μg/mL) and after 6 or 12 h, the cells were stained with our home-made kit or with a commercial kit from BD Biosciences (FITC Annexin Apoptosis Detection Kit II, BD Biosciences, San Diego, CA, USA). Cells were stained at a concentration of 10^5^ cells mL^−1^ in 100 μL of 10X Binding Buffer using 5 μL of PI and 5 μL of rhANXA5 conjugated with FITC (home-made kit) or 5 μL of annexin-FITC from FITC Annexin Apoptosis Detection Kit II. All data were collected in a FACSCanto II flow cytometer (BD Bioscience) and analyzed using FlowJo software (Tree Stat, San Carlos, CA, USA).

### Availability of supporting data

Supporting data are included in an additional file.

## Abbreviations

DOC: Dissolved oxygen concentration; FITC: Fluorescein isothiocyanate; HEPES: Hydroxyethylpiperazyne-N’-2-ethanesulfonic acid; LB: Lysogeny broth; MCB: Master cell bank; PI: Propidium iodide; PS: Phosphatidylserine; rhANXA5: Recombinant human annexin V; TB: Terrific broth.

## Competing interests

The authors declare that they have no competing interests.

## Authors’ contributions

LSM carried out part of the experiments and contributed to the manuscript writing. JL carried out part of the experiments and contributed to the manuscript writing. GR helped in fermentation and protein purification. DCR helped in cloning, amplification and purification. GOP generated the FITC-rhANXA5. JESN assisted in the production of rhANXA5 in the bioreactor. APDS conducted the FITC-annexin V binding experiments. ACOD helped in cloning and expression. CVB performed the identification and molecular mass determination of rhANXA5 by mass spectrometry and contributed to the manuscript writing. JMC and LAB assisted in most of the experiments and DSS devised the experiments. All authors read and approved the final manuscript.

## Supplementary Material

Additional file 1: Figure S1 SDS-PAGE analysis (12%) of samples from shaker cultivations of *Escherichia coli* BL21(DE3) and C41(DE3) strains in lysogeny broth (LB) and in our semi-defined (SD) media. M - Thermo ScientificTM Unstained Protein MW Marker; Lanes 1 and 5 - pET-30a(+) (empty vector) without IPTG induction; lanes 2 and 6 - pET-30a (+)::ANXA5 without IPTG induction; lanes 3 and 7 - pET-30a (+) (empty vector) after 6h of IPTG induction; lane 4 - pET-30a(+)::ANXA5 after 3h of IPTG induction; lane 8 - pET-30a (+)::ANXA5 after 6 h of IPTG induction. **a)** C41(DE3) and LB. Overexpression of pET-30a (+)::ANXA5 in E. coli C41(DE3) strain using LB media. **b)** BL21(DE3) and LB. Overexpression of pET-30a (+)::ANXA5 in E. coli BL21(DE3) strain using LB media. **c)** C41(DE3) and SD. Overexpression of pET-30a (+)::ANXA5 in *E. coli* C41(DE3) strain using SD media. **d)** BL21(DE3) and SD. Overexpression of pET-30a (+)::ANXA5 in *E. coli* BL21(DE3) strain using SD media. **Figure S2.** Densitometric analysis of lanes 4 and 8 from Figure S1d. **Figure S3.** rhANXA5 purification in MonoQ HR16/10 column. **(a)** Eluted fractions from MonoQ HR16/10 were analysed by SDS-PAGE (12%). M corresponds to Unstained Protein MW Marker (Fermentas); Lane 1 corresponds to crude extract; Lanes 2–12 corresponds to MonoQ HR16/10 elution fractions. **(b)** Chromatogram of eluted fractions. rhANXA5 was eluted in fractions 118 to 125 (inlet). **Figure S4.** SDS-PAGE densitometric analysis of rhANXA5 expression in fed-batch cultivations. M: Thermo Scientific™ Unstained Protein MW Marker; Lanes 1–3: samples collected at 30 h of culture (triplicate). rhANXA5 corresponded to (1) 45.3%, (2) 38.6% and (3) 37.9% of total protein content. Click here for file
