# Supplementary figures and images for: Production of recombinant human annexin V by fed-batch cultivation
Source: BMC Biotechnol. 2014 Apr 27;14:33. doi: 10.1186/1472-6750-14-33 (PMC4029966; doi:10.1186/1472-6750-14-33)

**SUPPLEMENTARY INFORMATION**

**Figure S1**


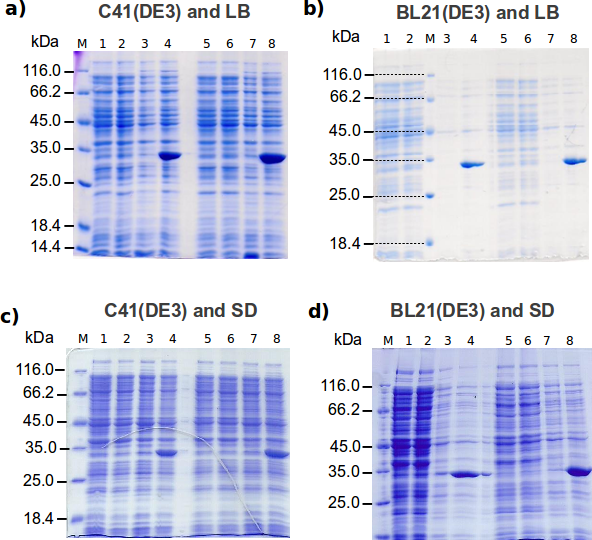


**Figure S2**

**
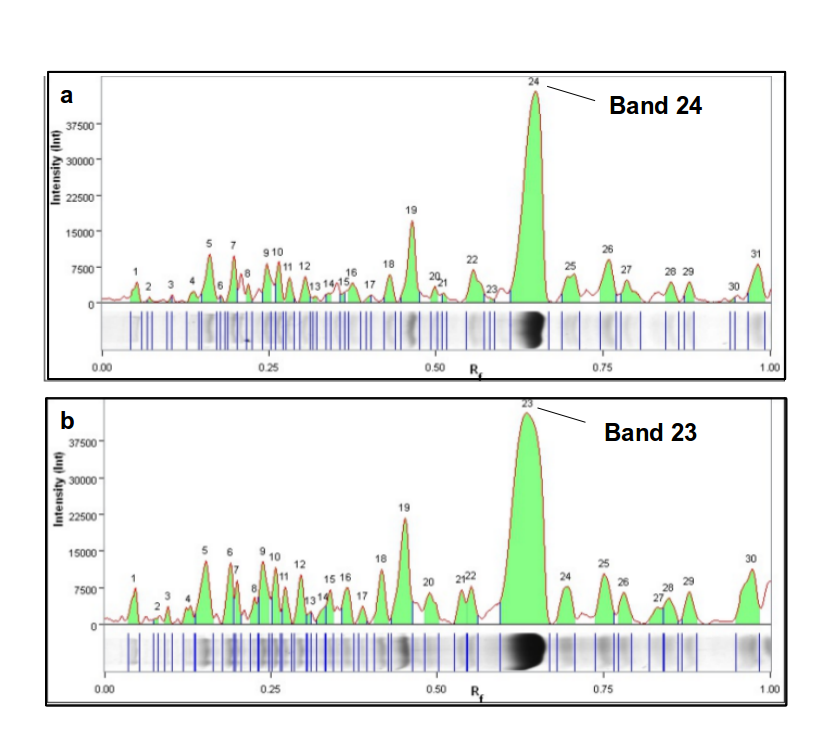
**


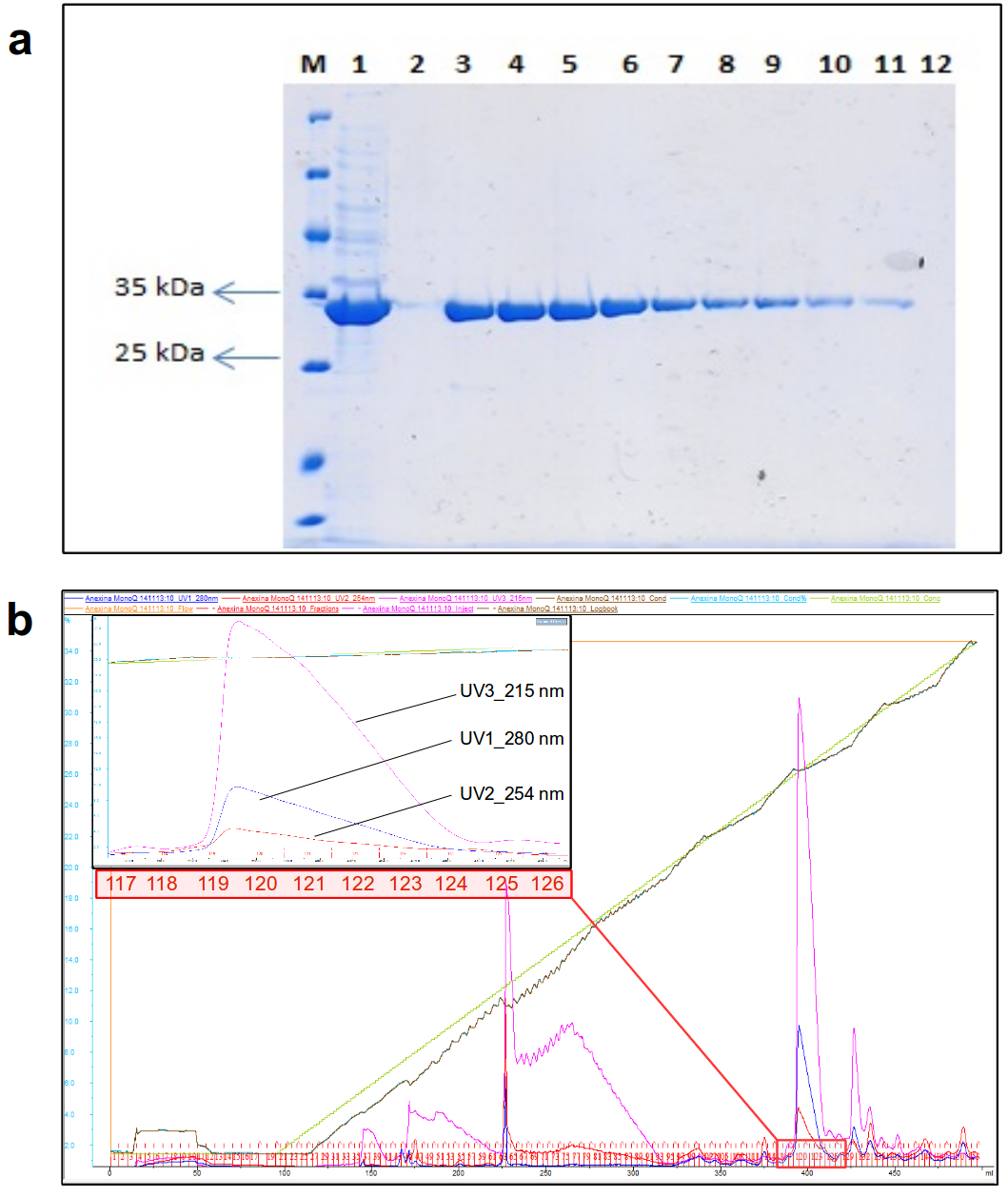
**Figure S3**

**Figure S4**


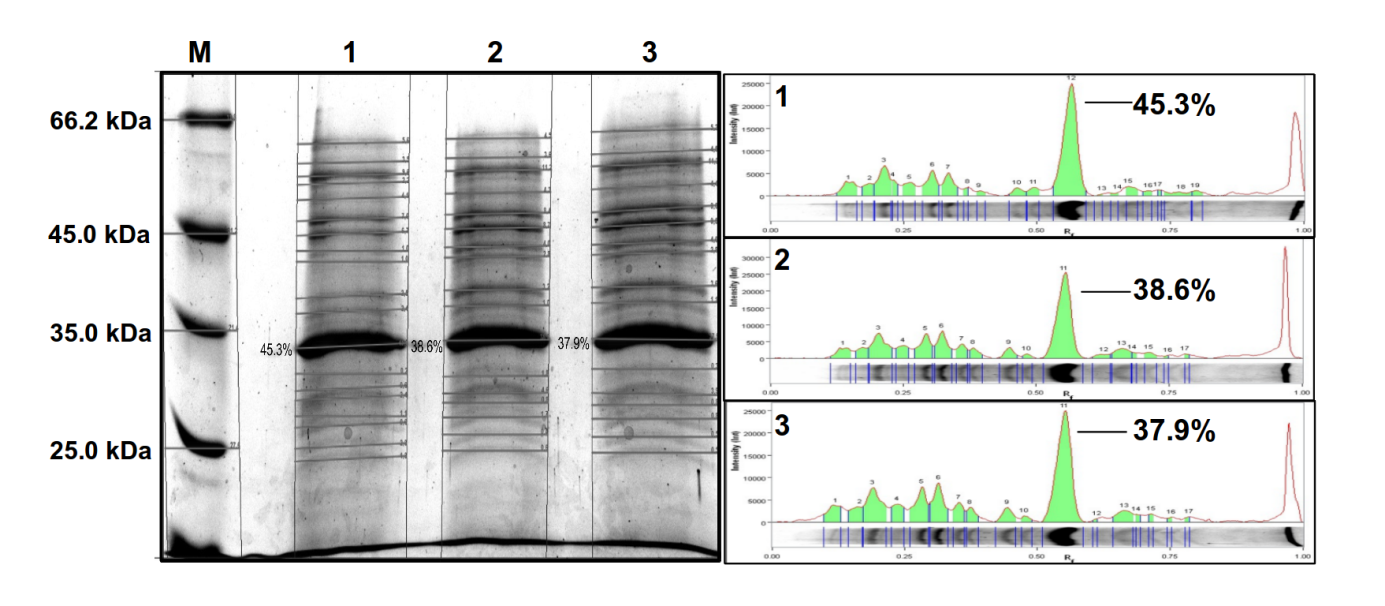

Supplement: Additional file 1: Figure S1 — SDS-PAGE analysis (12%) of samples from shaker cultivations of Escherichia coli BL21(DE3) and C41(DE3) strains in lysogeny broth (LB) and in our semi-defined (SD) media. M - Thermo ScientificTM Unstained Protein MW Marker; Lanes 1 and 5 - pET-30a(+) (empty vector) without IPTG induction; lanes 2 and 6 - pET-30a (+)::ANXA5 without IPTG induction; lanes 3 and 7 - pET-30a (+) (empty vector) after 6h of IPTG induction; lane 4 - pET-30a(+)::ANXA5 after 3h of IPTG induction; lane 8 - pET-30a (+)::ANXA5 after 6 h of IPTG induction. a) C41(DE3) and LB. Overexpression of pET-30a (+)::ANXA5 in E. coli C41(DE3) strain using LB media. b) BL21(DE3) and LB. Overexpression of pET-30a (+)::ANXA5 in E. coli BL21(DE3) strain using LB media. c) C41(DE3) and SD. Overexpression of pET-30a (+)::ANXA5 in E. coli C41(DE3) strain using SD media. d) BL21(DE3) and SD. Overexpression of pET-30a (+)::ANXA5 in E. coli BL21(DE3) strain using SD media. Figure S2. Densitometric analysis of lanes 4 and 8 from Figure S1d. Figure S3. rhANXA5 purification in MonoQ HR16/10 column. (a) Eluted fractions from MonoQ HR16/10 were analysed by SDS-PAGE (12%). M corresponds to Unstained Protein MW Marker (Fermentas); Lane 1 corresponds to crude extract; Lanes 2–12 corresponds to MonoQ HR16/10 elution fractions. (b) Chromatogram of eluted fractions. rhANXA5 was eluted in fractions 118 to 125 (inlet). Figure S4. SDS-PAGE densitometric analysis of rhANXA5 expression in fed-batch cultivations. M: Thermo Scientific™ Unstained Protein MW Marker; Lanes 1–3: samples collected at 30 h of culture (triplicate). rhANXA5 corresponded to (1) 45.3%, (2) 38.6% and (3) 37.9% of total protein content. [file 1472-6750-14-33-S1.doc]
